# Cost-effectiveness analysis of OM-85 vs placebo in the prevention of acute respiratory tract infections (ARTIs) in children that attend day-care centers

**DOI:** 10.1186/s13561-019-0230-1

**Published:** 2019-05-07

**Authors:** Arturo Berber, Blanca Estela Del-Rio-Navarro

**Affiliations:** 1 Fundación para el Avance de la Ciencia, Oasis 14, CP 02080 Mexico City, Mexico; 2 Allergy & Immunology Service, Hospital Infantil de Mexico “Federico Gomez”, Dr. Marquez 162, CP 06720 Mexico City, Mexico

**Keywords:** Acute respiratory tract infection, Prevention, Day-care-center, Immunostimulant, OM-85

## Abstract

**Background:**

Children that attend day-care centers frequently contract acute respiratory tract infections (ARTIs). ARTIs represent a burden for both children and parents. Systematic reviews on the use of immunostimulants for the prevention of juvenile recurrent ARTIs have provided moderate evidence of efficacy and safety. The aim of the study was to establish whether the immunostimulant, OM-85, was cost-effective in preventing ARTIs in children 2–6 years old that attended day-care centers or preschools in Mexico. We performed a systematic review to evaluate the efficacy of OM-85. For costs, we assumed an institutional perspective, which included the costs of care and supplies over a study period of six months, during the autumn-winter seasons. We created decision trees and constructed a model to identify pharmacoeconomic parameters. We generated 1000 estimations with the bootstrap method to calculate descriptive statistics of pharmacoeconomic parameters. We evaluated cost-effectiveness compared to treatment without immunostimulants.

**Results:**

The mean (SD) incidences of ARTIs were 5.59 ± 0.29 without immunostimulants and 2.97 ± 0.32 with OM-85, during the study period. The mean (25th, 75th percentile) direct costs of ARTIs were 57.04 (37.11, 76.39) US$ (US dollars) without immunostimulants and 48.53 (37.35, 58.93) US$ with OM-85, with a mean increment of − 8.51(− 17.08, 0.75) US$, and a mean cost-effectiveness of − 17.94 (− 36.48, 1.66) US$. The direct costs plus the cost of one parent missing work to care for the child with ARTI were 125.76 (102.83, 150.16) US$, without immunostimulant and 85.21 (72.15, 98.81) US$, with OM-85. The increment was − 40.55 (− 68.29, − 13.95) US$, and the cost-effectiveness was − 86.89 (− 142.37, − 29.34) US$.Part of the cost reduction was ascribed to the reduced use of medications, particularly antibiotics.

**Conclusions:**

Our results were consistent with previous clinical studies conducted in closed institutions in Mexico. OM-85 reduced the number of ARTIs and the frequency of antibiotics use. We concluded that OM-85 was cost-effective for preventing ARTIs in children that attended day-care centers, particularly when parental absenteeism was covered by the institutions.

## Background

Children that attend day-care centers are at increased risk of contracting an acute respiratory tract infection (ARTI). A systematic review on the children that attend day-care centers found elevations in the relative risk (RR) of upper ARTIs (RR: 1.88), acute otitis media (RR: 1.58), and lower ARTIs (RR: 2.10) [1]. For instance, in a Mexican study of children aged 1.5 to 4 months, the ARTI incidence was 6 episodes per year, with a median of 40 sick-days, in those staying at home; in contrast, children in day-care centers experienced 14 ARTIs per year, with 74 sick-days [2]. Additionally, the cost of ARTI treatments was calculated to be twice as high for children in day-care centers as the cost for children not in day-care centers [3]. Similarly, in Chile, for a cohort of children attending a day-care center, the total cost per ARTI was US$ 129.00 for infants and US$ 53.00 for toddlers; the cost contributed by parents missing work to care for a sick child (parental job absenteeism) were US$ 105.00 (81% of the total cost), for infants, and US$ 38.00 (71% of the total cost), for toddlers [4].

OM-85 is a lyophilized bacterial lysate that comprises 21 bacterial strains used in the prevention of ARTIs. OM-85 was previously tested in twelve double-blind placebo control clinical trials in children [5–16]; of those trials, seven showed efficacy, where the effect was expressed as the percentage difference in the number of ARTIs compared to placebo [5, 7, 9, 10, 12, 13, 16]. A Cochrane systematic review found that OM-85 had an effect of − 35.90% (range: − 49.46, − 22.35%) [17]. Their meta-analysis included randomized controlled trials (RCTs) and compared the ability of immunostimulants, administered by any method, to prevent ARTIs, compared to placebo. Trial participants were under 18 years of age with no allergic or chronic conditions. The studied outcomes comprised the differences between groups in the number of ARTIs, the percentage of ARTIs, and the incidence of adverse events in children. The submeta-analysis of OM-85 [16] included studies by Ahrens (1984) [5], Del-Rio-Navarro (2003) [7], Gomez-Barreto (1998) [8], Gutierrez-Tarango (2001) [9], Jara-Perez (2000) [10], Maestroni (1984) [11], Schaad (1986) [14], Schaad (2002) [15], and Zagar (1988) [16].

Another meta-analysis that considered only Mexican studies (Del-Rio-Navarro (2003) [7], Gomez-Barreto (1998) [8], Gutierrez-Tarango (2001) [9], and Jara-Perez (2000) [10]) calculated an effect of − 46.85% (− 54.98, − 38.72) [18]. This same systematic review reported that immunostimulants for ARTI prevention in children caused gastrointestinal adverse events, with a global incidence of 30 per 1000 treated children (95% confidence interval (CI): 11, 50), and skin adverse events, with an incidence of 7 per 1000 (95% CI: -8, 14) [17].

### Study objective

We aimed to establish whether the immunostimulant, OM-85 compared to placebo, was cost-effective for the prevention of ARTIs in preschool-age children (aged 2 to 6 years) that attended day-care centers and preschools in Mexico. We included children that had experienced six or more ARTIs in the prior 12 months, because less than six ARTIs per year is considered normal in children [1–4].

### Study hypothesis

We hypothesized that the immunostimulant, OM-85, would be cost-effective in the prevention of ARTIs in susceptible preschool-age children that attended day-care centers or preschools in Mexico.

### Study perspective

We assumed an institutional perspective, where the institution was responsible for patient medical care, medications, and the salary of parents that missed days of work to attend to their sick children. When a child is sick, he/she is not allowed to attend the day-care-center/preschool. One of the parents (mainly mothers) must take to the child to a social medicine clinic to obtain medical care and a certificate of permission to be absent from the job while taking care of the child. The total earnings pertaining to the absent work days are covered by the institution.

### Time horizon; study period

The study period covered six months during the fall and winter seasons of 2017–2018 as it explained the most of ARTIs in the year. Discount rate was not applied, because the short period.

The children were followed for six months, starting at the beginning of the fall season and the initiation of the immunostimulant treatment. The modeling period corresponded to the period that the immunostimulant was expected to provide the protective effect demonstrated in clinical trials. It covered the time interval associated with the highest ARTI incidence; 60 to 70% of all ARTIs occurred in the fall and winter seasons (expert panel).

## Methods

We created hypothetical patients, based on the incidence and costs of children that attended day-care-centers/preschools reported by the panel of experts. The incidence of ARTIs and the costs of treatment are described below.

We collected data on the incidence of ARTIs, the kind of ARTIs, and the treatment costs from a group of physicians assigned to the Hospital Infantil de México, “Federico Gómez”; All costs are expressed in Mexican pesos ($MXN), valued in October 2017. The exchange rate was $20.00 pesos per one American dollar. The purchase power parity (PPP) reported for $MXN in 2017 was 9.041 (https://data.oecd.org/conversion/purchasing-power-parities-ppp.htm).

We next used these data to elaborate two decision trees, as they best reflected the current occurrence of the ARTIs in the population. The first included the probabilities and number of ARTIs in the group of interest; the second included the probabilities of the different kinds of ARTIs and their treatment costs. We determined the efficacy of OM-85 by performing a meta-analysis of the double-blind, placebo-controlled trials conducted in Mexico. We also considered other data, including the cost range of ARTI treatments, which included the OM-85 doses, parental absenteeism, and treatments for adverse events. A cost model was used to calculate the cost, cost effectiveness, and incremental cost-effectiveness. We employed the bootstrap method to create 1000 replications for a group of 2000 subjects, based on the ranges of probability for each event and the implied range of costs. Bootstrap estimations were used to calculate the mean, median, range, and quartiles of pharmacoeconomic parameters. The results were plotted to evaluate cost-effectiveness (scatter-plots; incremental cost vs. incremental effectiveness plots show the mean differences in the costs and outcomes of OM-85 treatment compared to typical treatments) and cost-effectiveness vs. acceptability (curves; probability of cost-effectiveness vs. willingness to pay curves show the probability that OM-85 treatment would be cost-effective, based on how much the payer is willing to pay).

## Results

Table 1 shows the annual frequency of upper and lower ARTIs in children aged 2 to 6 years that attended day-care centers or preschools, according to data acquired from the expert panel (including five pediatric allergists and five pediatric otorhinolaryngologists that worked at the Hospital Infantil de Mexico Federico Gomez and also practiced in private offices). The different diagnoses and their respective treatments were described by the expert panel. The expert panel estimated that 60 to 70% of all ARTIs occurred in the fall and winter seasons. In the population of interest, children with ARTIs spent 5 to 7 days out of day care/preschool, and one of the parents was allowed to take days off work to care for their ill children; the full salary that accrued while the parent cared for the child was covered by the institution.

**Table 1:** Frequency of upper and lower acute respiratory tract infections (ARTIs), specific diagnoses, and corresponding treatment, in pediatric patients that attended day-care centers or preschool estimated by the expert panel

ARTI parameters	Frequency	Treated without Antibiotics	Treated with Antibiotics
Upper ARTIs (number per year)			
< 3	10%	10–30%	70–90%
3 to 6	30–50%	10–30%	70–90%
6 to 10	50–80%	10–30%	70–90%
> 10	10%	10–30%	70–90%
Lower ARTIs (number per year)			
1	97%	0%	100%
2 to 3	1%	0%	100%
4 to 6	1%	0%	100%
6 to 10	1%	0%	100%
Type of upper ARTI			
Simple upper ARTI (Common cold, rhinopharyngitis, tonsillitis, pharyngotonsillitis)	70%	20–30%	70–80%
Otitis media	25%	20%	80%
Rhinosinusitis	5%	10%	90%

The estimated costs for the ARTI treatments, without and with antibiotics, are shown in Table 2.

**Table 2:** Cost of one treatment for acute respiratory tract infection, without or with antibiotics estimated by the expert panel

Medication	Age: Treatment time (patient weight)	Treatment cost ($MXN)
Treatments without antibiotics		
Antihistamine combinations		
Antifludes™	2–6 years: 3–5 days	$68.95
Sensibit™	2–6 years: 3–5 days	$202.50–$79.50
LM6™	2–6 years: 3–5 days	$69.00
Antipyretics/ Anti-inflammatory drugs		
Ibuprofen	2 years: 3–5 days	$79.50–$34.50
	4 years: 3–5 days	$ 159.00-$69.00
	6 years: 3–5 days	$ 159.00-$69.00
Paracetamol	2 years: 3–5 days	$80.00–$34.50
	4 years: 3–5 days	$ 80.00-$34.50
	6 years: 3–5 days	$ 160.00-$69.00
Nimesulide	2 years: 3–5 days	$223.50–$86.95
	4 years: 3–5 days	$ 223.50-$86.95
	6 years: 3–5 days	$ 447.00-$173.90
Treatment with antibiotics		
Amoxicillin	2 years: 7–10 days (12.5 kg)	$199.00–$61.95
	4 years:7–10 days (16 kg)	$199.00–$61.95
	6 years: 7–10 days (21 kg)	$298.50–$123.90
Amoxicillin + Clavulanic acid	2 years: 7–10 days (12.5 kg)	$250.95–$85.95
	4 years:7–10 days (16 kg)	$250.95–$171.90
	6 years: 7–10 days (21 kg)	$501.90–$171.90
Cefuroxime	2 years: 7–10 days (12.5 kg)	$520.5–275.90
	4 years:7–10 days (16 kg)	$1041.00–275.90
	6 years: 7–10 days (21 kg)	$1041.00–413.85
Cefalexin	2 years: 7–10 days (12.5 kg)	$655.00–$159.90
	4 years:7–10 days (16 kg)	$655.00–$159.90
	6 years: 7–10 days (21 kg)	$982.5–$239.85
Clarithromycin	2 years: 7–10 days (12.5 kg)	$494.95–$241.40
	4 years:7–10 days (16 kg)	$494.95–$241.50
	6 years: 7–10 days (21 kg)	$989.9–$483.00
Cephalosporin (3rd Generation)	2 years: 7–10 days (12.5 kg)	$486.50–$514.00
	4 years:7–10 days (16 kg)	$637.00–$685.95
	6 years: 7–10 days (21 kg)	$637.00–$685.95

The lowest and highest estimates for direct costs for one ARTI treated without antibiotics were 95.00 $MXN (Mexican pesos) and 1450.00 $MXN, respectively; the corresponding costs for a treatment with antibiotics were 157.00 $MXN and 2491.00 $MXN, respectively. The cost of one parent missing work for one ARTI ranged from 630.00 $MXN to 2100.00 $MXN. Pediatric OM-85 (10 capsules, 3.5 mg each) cost 430 $MXN at the drugstore. The complete treatment consisted of 30 capsules; thus, the total cost was 1290 $MXN.

We also investigated adverse events. The Cochrane systematic review indicated that the use of immunostimulants to prevent ARTIs was associated with 30 gastrointestinal and 7 dermic events per 1000 treated patients. We assigned a mean cost of 500.00 $MXN per adverse event.

**Fig. 1:**
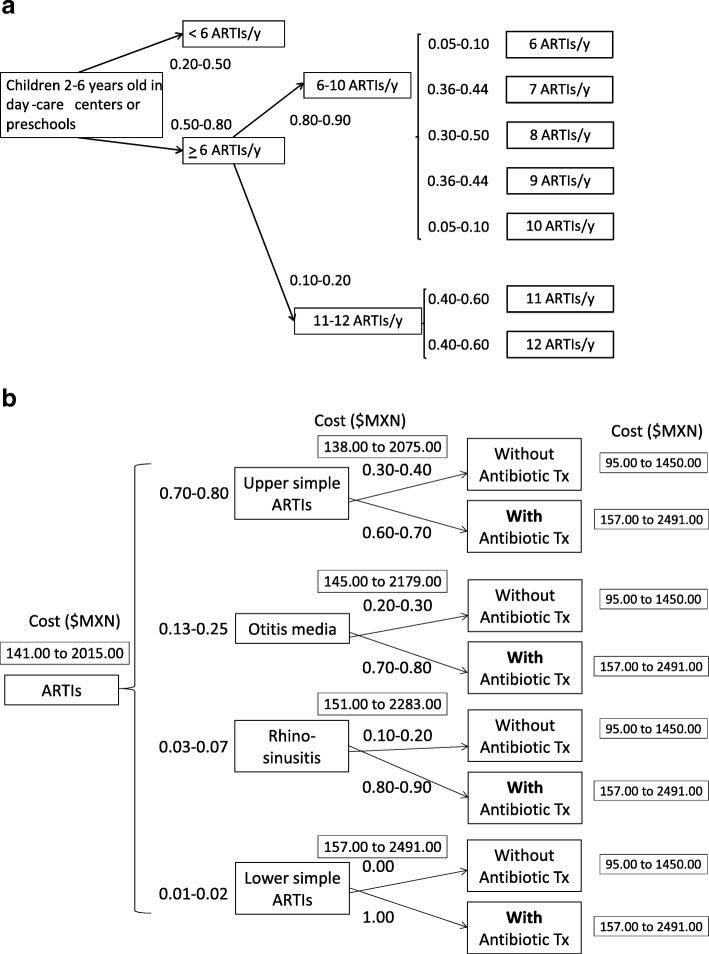
Decision tree analyses for frequencies and types of ARTIs and the typical treatments in 2–6 year-old children that attended day-care centers or preschools. (a) Frequency of ARTIs; (b) differential diagnosis of ARTIs, their treatments, and the costs of treatment. ARTI: acute respiratory tract infection; Tx: treatment

Figure 1 shows the decision tree analyses of ARTI frequencies, types, and typical treatments. With these values, we calculated the ranges of probabilities and costs, which were used to generate 1000 estimations with the bootstrap method (Table 3).

**Table 3:** Probabilities of ARTIs in 2–6 year-old children at day-care centers or preschools and estimated costs, for use in the bootstrap method estimated by the expert panel

Event	Range
≥6 ARTIs/y	0.50–0.80
6–10 ARTIs/y	0.80–0.90
11–12 ARTIs/y	0.10–0.20
6 ARTIs/y	0.05–0.10
7 ARTIs/y	0.36–0.44
8 ARTIs/y	0.30–0.50
9 ARTIs/y	0.36–0.44
10 ARTIs/y	0.05–0.10
11 ARTIs/y	0.40–0.60
12 ARTIs/y	0.40–0.60
ARTIs in Fall-Winter Season	0.60–0.70
Simple acute lower ARTIs	0.01–0.02
Simple acute upper ARTIs	0.70–0.80
Acute otitis media	0.13–0.25
Acute rhinosinusitis	0.03–0.07
Tx with antibiotics for simple acute lower ARTIs	1
Tx without antibiotics for simple acute upper ARTIs	0.30–0.40
Tx with antibiotics for simple acute upper ARTIs	0.60–0.70
Tx without antibiotics for acute otitis media	0.20–0.30
Tx with antibiotics for acute otitis media	0.70–0.80
Tx without antibiotics for acute rhinosinusitis	0.10–0.20
Tx with antibiotics for acute rhinosinusitis	0.80–0.90
Cost of Tx without antibiotics ($MXN)	95–1450
Cost of Tx with antibiotics ($MXN)	157–2491
Cost of parent job absenteeism ($MXN)	630–2100
OM-85 effectiveness (ARTI reduction)	0.38–0.55
Cost of OM-85 ($MXN)	258–430
Cost of OM-85 adverse events ($MXN)	11.1–22.2

Values are frequencies, unless otherwise indicated. *ARTI* acute respiratory tract infection, *Tx* treatment

The mean (± SD) incidences of ARTIs were 5.59 ± 0.29 without immunostimulants and 2.97 ± 0.32 with OM-85, during the study period. The mean (25th, 75th percentile) direct costs of ARTIs were 1140.80 (742.10, 1527.80) MXN$ [57.04 (37.11, 76.39) US$; 515.70 (335.47, 690.64) $MXN adjusted by PPP] without immunostimulants and 970.60 (746.90, 1178.50) $MXN [48.53 (37.35, 58.93) US$; 438.76 (337.64; 532.74) $MXN adjusted by PPP] with OM-85, with a mean increment of − 170.20 (− 341.50, 14.90) $MXN [− 8.51(− 17.08, 0.75) US$; − 76.94 (− 154.38, 6.74) $MXN adjusted by PPP], and a mean cost-effectiveness of − 358.80 (− 729.50, 33.10) $MXN. [− 17.94 (− 36.48, 1.66) US$; − 162.20 (− 329.77, 14.96) $MXN adjusted by PPP]. Part of the cost reduction was ascribed to the reduced use of medications, particularly antibiotics. The direct costs plus the cost of one parent missing work to care for the child with ARTI were 2515.10 (2056.50, 3003.10) $MXN [125.76 (102.83, 150.16) US$; 1136.95 (929.64, 1357.55) $MXN adjusted by PPP] without immunostimulant and 1704.10 (1442.90, 1976.20) $MXN [85.21 (72.15, 98.81) US$; 770.34 (652.26, 893.34) $MXN adjusted by PPP] with OM-85. The increment was − 811.00 (− 1365.80, − 279.00) $MXN [− 40.55 (− 68.29, − 13.95) US$; − 366.61 (− 617.41, − 126.12) $MXN adjusted by PPP], and the cost-effectiveness was − 1737.80 (− 2847.30, − 586.70) $MXN.[− 86.89 (− 142.37, − 29.34) US$; − 785.57 (− 1287.12, − 265.22) $MXN adjusted by PPP]. In all conditions, OM-85 showed cost-effectiveness. Moreover, the OM-85 group showed cost savings in over 70% of cases for direct costs (See Table 4).

**Table 4:** Descriptive statistics for the pharmacoeconomic variables used in this study with data from meta-analysis and estimated by the expert panel

Assessment	Mean	SEM	Median	SD	Variance	Min	Max	25th Pctl	75th Pctl
OM-85									
Effectiveness	0.466	0.002	0.467	0.049	0.002	0.38	0.55	0.424	0.508
Tx without IS, n	5.6	0.01	5.59	0.29	0.09	4.93	6.3	5.37	5.85
Tx with OM-85, n	2.99	0.01	2.97	0.32	0.1	2.31	3.83	2.74	3.24
Incremental ARTIs, n	−2.61	0.01	−2.6	0.31	0.09	−3.4	−1.9	−2.82	− 2.38
Direct Costs ($MXN)									
Tx without IS	1140.8	15.1	1124.2	478.7	229,163	180	2149.1	742.1	1527.8
Tx with OM-85	970.6	8.5	965.1	268.8	72,235	370.6	1647.9	746.9	1178.5
Incremental	−170.2	7.4	−154.6	235.1	55,282.5	− 839.4	316.1	−341.5	14.9
Cost-Effectiveness	−358.8	15.6	− 326.7	493.1	243,194	− 1528.9	756.2	−729.5	33.1
Direct Cost + Absenteeism ($MXN)									
Tx without IS	2515.1	20.5	2495.4	648.1	419,984	944.5	4140.4	2056.5	3003.1
Tx with OM-85	1704.1	11.8	1681.2	373	139,107	853.9	2758.8	1442.9	1976.2
Incremental	−811	23.3	− 790	737.8	544,411	− 2722.1	1490.8	−1365.8	−279
Cost-Effectiveness	−1737.8	50.8	− 1662.5	1606.4	2,580,387	− 6705.7	3566.4	−2847.3	−586.7

Absenteeism, parent missing work to care for a sick child; *ARTIs* acute respiratory tract infections, *IS* immunostimulant, *Max* maximum, *Min* minimum, *Pctl* percentile, *SD* standard deviation, *SEM* standard error of the mean, *Tx* treatment for acute respiratory tract infection

**Fig. 2:**
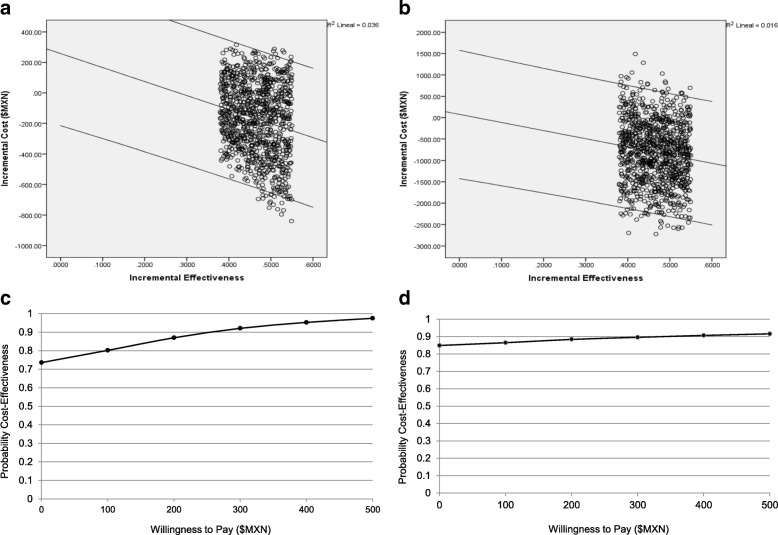
Cost-effectiveness and acceptability of OM-85 treatment for acute respiratory tract infection. (**a**, **b**) Incremental cost vs. incremental effectiveness plots show the mean differences in the costs and outcomes of OM-85 treatment compared to typical treatments, based on data from 1000 bootstrap replicates. Negative costs represent savings with OM-85 compared to typical treatments. (**a**) Direct costs; (**b**) direct costs plus parental absenteeism cost (i.e., the cost of one parent missing work to care for the child). Lines show the mean and 95% confidence intervals; (**c**, **d**) Probability of cost-effectiveness vs. willingness to pay curves show the probability that OM-85 treatment would be cost-effective, based on how much the payer is willing to pay. (**c)** Direct costs; (**d**) direct costs plus parental absenteeism cost

Figure 2 shows cost-effectiveness scatter-plot and cost-effectiveness acceptability curves, both for direct costs and direct costs plus absenteeism cost. The cost-effectiveness scatter-plot for direct costs showed that OM-85 provided positive incremental effectiveness for all ARTI conditions. Moreover, OM-85 provided cost savings (negative incremental costs) for more than 70% of conditions. The cost-effectiveness scatter-plot for the direct costs plus the parent absenteeism cost showed that OM-85 provided positive incremental effectiveness for all ARTI conditions, and in 90% of ARTI conditions, OM-85 provided cost savings.

The acceptability curve of direct costs showed that the probability of achieving cost-effectiveness was above 70%, even when the willingness to pay was zero; it reached 80%, when the willingness to pay was between 100.00 and 150.00 $MXN. Moreover, the acceptability curve of direct costs plus absenteeism cost showed that the probability of cost-effectiveness was above 80%, when the willingness to pay was zero, and it rose to 90%, when the willingness to pay was 250.00 $MXN.

## Discussion

The use of immunostimulants for the prevention of ARTIs in children has been controversial for several years [19]. Yet, systematic reviews [17–19] have supported their effectiveness with evidence of moderate quality. It was shown that immunostimulants could reduce ARTI frequency by nearly 40%, but they also provoked secondary effects, with an adverse event rate (gastrointestinal and dermic events) of 37/1000 treated patients [17].

Previous pharmacoeconomic evaluations have studied immunostimulants for preventing pediatric ARTIs [20–22]. For example, Pessey 2003 [20] evaluated the pharmacoeconomic value of using OM-85 for the prevention of rhinopharyngitis in French children, based on the low efficacy reported in three European studies [11, 13, 16] and the cost structure of the French Social Security system. They performed a sensitivity analysis with extreme values, in a model constructed without generating a cloud of estimations. According to their study, the cost of one episode of acute rhinosinusitis was €49.39 (value in the year 2000) and the use of OM-85 would prevent 1.52 infections over six months, with a savings of €67.83 (range: €6.28 to €303.64). They concluded that OM-85 was a cost-effective intervention.

Another study, based on conditions in Italy [21], found that a complete cycle of OM-85 treatment could reduce the number of upper ARTIs by 1.60/pediatric patient (ages 6 months to 19 years) over six months. This reduction in upper ARTIs could save €107.42/patient, from the family’s perspective; €231.26, from the community perspective; and €48.52, from the National Health System perspective. They estimated that the use of OM-85 in ARTI prevention would be cost-effective, when more than 7% of upper ARTIs were prevented and the total cost of treating one upper ARTI was greater than €10.00. Another Italian pharmacoeconomic evaluation [22] estimated that a complete course of OM-85 would reduce the number of ARTIs by 1.2/pediatric patient in a six-month period. This would achieve a savings of €40.30/patient (2015 prices), from the perspective of the National Health Service, and the savings would be €182.99/patient, from the perspective of the community.

The present study differed from previous studies in several points. We investigated the immunostimulant effect on day-care-center/preschool children, a population at high risk of contracting ARTIs. We focused on patients with six or more ARTIs in the 12 months prior to the study. The study took into account the incidence of the different kinds of ARTIs, their typical treatments, and the corresponding costs. However, the present study did not include extra costs for laboratory tests and other procedures, because they are not typically ordered for this type of patient. We employed a cloud of 1000 estimations, which allowed a better sensitivity analysis.

The main limitation of the present study was that the data on ARTI frequencies, types, typical treatments, and costs were obtained from a panel of pediatricians that worked in a public hospital and also in private practice. Alternative means for retrieving this information might be to compile information from medical charts or to perform a formal field study.

The present study focused on the individual impact of ARTI prevention from the institutional point of view. Future clinical studies and pharmacoeconomic evaluations should include the implications for day-care centers and preschools; they should include both the community and individual points of view; and they should include the impact of the results on the National Health System.

The results of the present pharmacoeconomic evaluation were consistent with previous observations found in an open study of orphan homes in Baja California, Mexico [23] and in a double-blind placebo-controlled study conducted in the Girl’s Home in Mexico City [10]. Those studies also reported that immunostimulant treatments reduced the number of ARTIs in treated participants and reduced the use of medications, particularly antibiotics.

## Conclusions

We found that the immunostimulant, OM-85, was a cost-effective intervention for the prevention ARTIs in high risk children, aged 2 to 6 years, that attended day-care centers or preschools. This intervention was particularly cost-effective, when the costs of parental job absenteeism were also considered.

## Abbreviations

$MXN: Mexican pesos
€: Euros
ARTI: Acute respiratory tract infection
CI: Confidence interval
RR: Relative risk
SD: standard deviation
US$: USA dollars
